# Advantages and effectiveness of AI three-dimensional reconstruction technology in the preoperative planning of total hip arthroplasty

**DOI:** 10.1038/s41598-025-09488-9

**Published:** 2025-07-09

**Authors:** Shulin Li, Jilin Jiang, Jiahao Zhang, Laipeng Yan, Huiling Guo, Faqiang Tang

**Affiliations:** 1 Shengli Clinical Medical College of Fujian Medical University, Fuzhou, China; 2 Department of Orthopedics, Fujian Provincial Hospital, Fuzhou, China; 3 Fuzhou University Affiliated Provincial Hospital, Fuzhou, China; 4 College of Integrative Medicine, Fujian University of Traditional Chinese Medicine, Fuzhou, China

**Keywords:** Outcomes research, Clinical trials

## Abstract

In order to explore the application effect of artificial intelligence (AI) 3D reconstruction technology in total hip arthroplasty (THA), this study included a total of 109 patients with unilateral femoral head ischemic necrosis. According to the preoperative planning method, they were divided into the AI group (n = 55) and the 2D group (n = 54). The operating time, intraoperative bleeding, length of hospital stay length, prosthesis conformity, imaging indicators and Harris scores. The complete conformity rates of the acetabular cup and femoral stem in the AI group (90.9% and 87.3%) were significantly higher than those in the 2D group (72.2% and 66.7%) (*P* < 0.05). The perioperative indicators of the AI group, such as operating time intraoperative bleeding volume and length of hospital stay were all better than those of the 2D group (*P* < 0.05). The AI group had significantly less postoperative leg length discrepancy (LLD) than the 2D group (*P* < 0.05). The Harris score at 1 month, 3 months and 6 months after surgery was significantly higher in the AI group than in the 2D group, and the difference was statistically significant (*P* < 0.05). Using AI 3D reconstruction technology to perform preoperative planning for patients scheduled to undergo THA can assist clinicians in completing the surgery more quickly and accurately, effectively control the patient’s postoperative LLD, and also reduce intraoperative bleeding, shorten the patient’s hospital stay, and accelerate the patient’s postoperative functional recovery.

## Introduction

Total hip arthroplasty (THA) is one of the most effective procedures for improving end-stage hip disease. However, when the prosthesis is not properly selected or the components are poorly placed, it can lead to a series of intraoperative and postoperative complications, such as pain, leg length discrepancy (LLD), stress shielding, joint dislocation, periprosthetic fracture and even aseptic loosening^1,2^. Therefore, successful THA requires selecting the most appropriate prosthesis type for the patient and placing it in the exact position to reestablish joint biomechanics as much as possible^3^. The most important thing is to choose the most appropriate type of prosthesis for the patient and to place it in the exact position to reconstruct the joint biomechanics as much as possible.

Accurate preoperative planning can help operators obtain information about the reconstruction outcome in advance and avoid poor component alignment to minimize complications. Currently, the most widely used preoperative planning method is two-dimensional (2D) X-ray film template measurement, but its accuracy is low because of the influence of factors such as the projection position and magnification when X-ray images are taken, as well as planning bias due to personal experience when subsequent template measurements are performed. Artificial intelligence (AI) has made significant progress in performing diagnostic tasks in medical imaging and, in particular, has demonstrated expert capabilities in musculoskeletal radiology, including accurate identification of specific orthopedic placement models^4,5^. This study aims to investigate the value of AI 3D reconstruction technology in THA by comparing the effectiveness of the 2D radiograph template measurement method with that of AI 3D reconstruction technology in the preoperative planning of THA.

## Methods

### General information

The study was approved by the Ethics Committee of Fujian Provincial Hospital to retrospectively search our hospital database, and finally included 109 patients with unilateral ischemic necrosis of the femoral head who received primary TKA for the first time at the Department of Orthopaedics of Fujian Provincial Hospital from September 2021 to September 2023. Among these patients, 55 cases were preoperatively planned via AI-HIP, version 1.0 (Beijing Changmugu Medical Technology Co., Ltd. https://www.changmugu.com/index/index/product.html) software (AI group), and 54 cases were preoperatively planned via 2D X-ray template measurements provided by DePuy Synthes (2D group). In the AI group, there were 26 males and 29 females, with a mean age of 63.16 ± 6.95 years and a mean BMI of 22.91 ± 3.63, in which 22 patients were in stage III and 33 patients in stage IV in Ficat. The difference of bilateral femoral eccentricity, LLD, and Harris score of hip joint function before operation were (6.67 ± 3.58) mm, (13.31 ± 5.63) mm, (6.89 ± 1.05) points, and (35.38 ± 8.73) points respectively. In the 2D group, there were 25 males and 29 females, with a mean age of 61.76 ± 9.65 years and a mean BMI of 23.33 ± 3.58, in which 23 patients were in stage III and 31 patients in stage IV in Ficat. The difference of bilateral femoral eccentricity, LLD, VAS score, and Harris score of hip joint function before operation were (6.60 ± 4.66) mm, (14.06 ± 6.51) mm, (6.65 ± 1.12) points, and (32.56 ± 9.23) points respectively. There was no significant difference in the baseline data between the two groups (*P* > 0.05).

All research conducted was performed in accordance with the relevant guidelines and regulations. As the study was a retrospective study without patient intrusion or intervention, all data were anonymized, so the author did not directly obtain consent from each patient. The need for informed consent was waived by the ethics committee of Fujian Provincial Hospital.

### Treatment

**Fig. 1:**
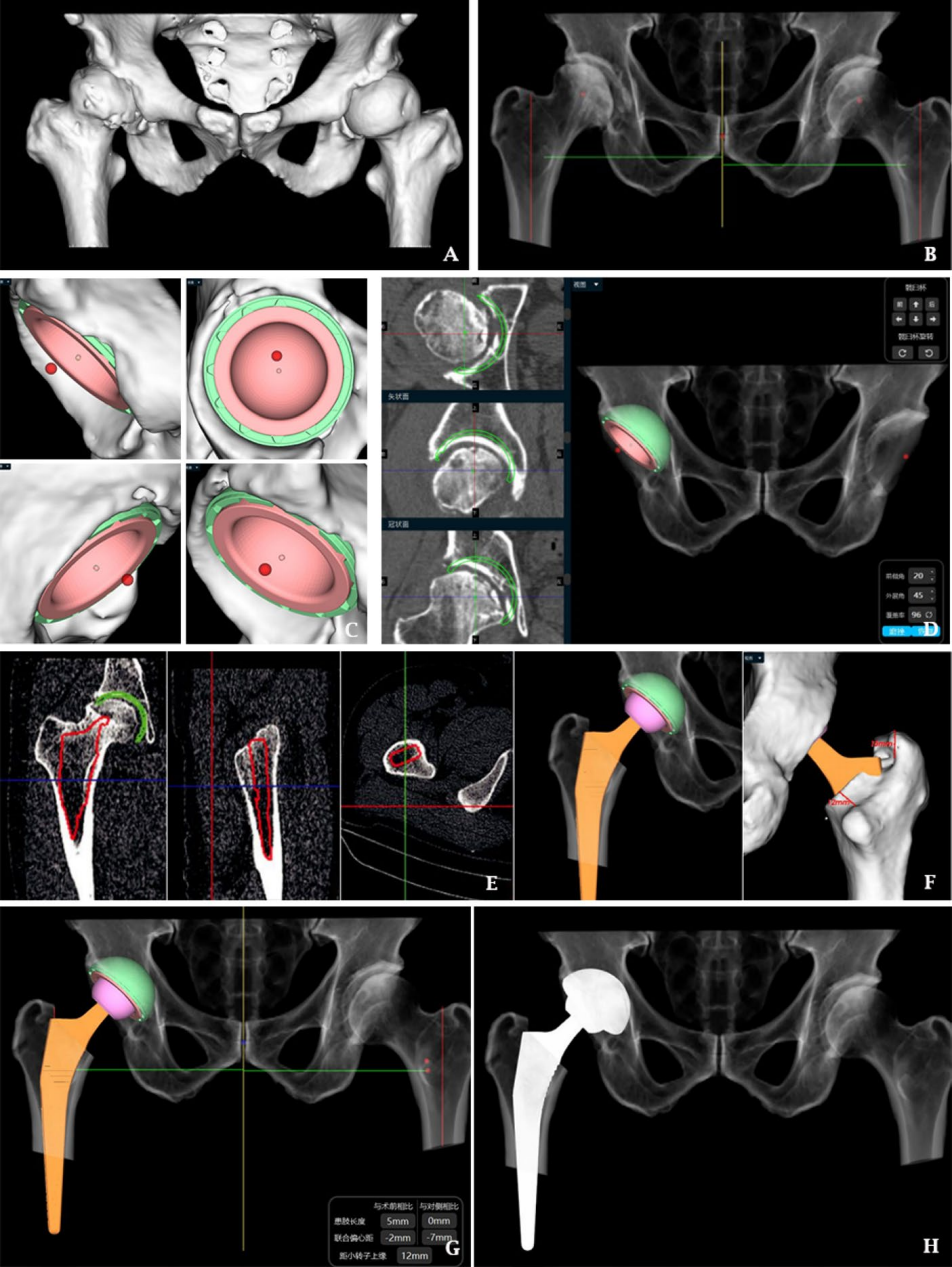
Schematic diagram of AI-HIP 3D preoperative planning.

In the AI group, bilateral CT scanning of the hip joint is performed before surgery, the data are exported in DICOM format to AI-HIP software, the system automatically recognizes the anterior superior iliac spine, pubic symphysis, tear drop, and greater and lesser trochanter of the femur, and the parameters of the acetabulum, the diameter of the femoral bone marrow cavity, and the angle of the femoral neck-trochanter are measured to generate three-dimensional reconstructive imaging; the system intelligently recognizes the acetabulum and femur via G-NET neural network technology to achieve accurate AI segmentation of the acetabulum and femoral head. The system uses G-NET neural network technology to identify the acetabulum and femur to achieve accurate segmentation of the acetabulum and femoral head by AI, obtain the acetabular rotation center, automatically identify the position and size of the acetabulum, and plan for the appropriate anterior inclination and abduction angle; at the same time, the system matches the appropriate femoral stem according to the femoral bone marrow cavity diameter and simulates osteotomy with reference to the prosthetic osteotomy line to ensure the restoration of the isometric length of the two lower limbs, ultimately generating the preoperative planning of AI and the simulation of postoperative X-rays (Fig. 1).

In the 2D group, preoperative orthopantomograms of the pelvis were obtained, and to obtain X-rays with standard magnification ratios, all patients were marked by placing a 1 cm diameter steel ball in the lateral thigh at the location of the flat macronodular node when the films were taken. Preoperative measurements were taken via a prosthetic film template provided by DePuy Synthes to complete preoperative planning^6^.

All surgeries were performed under general anesthesia in a 90° lateral position. The same surgical team performed the surgeries using the classic posterolateral approach. All joint prostheses were from DePuy Synthes. After the operation, the patient received functional rehabilitation guidance from the same rehabilitation team.

### Evaluation index

Comparisons of the operation time, intraoperative bleeding, hemoglobin difference, hospitalization time, the size of the prosthesis used in surgery, imaging indices (anterior tilt angle, abduction angle, LLD and bilateral femoral eccentricity difference)^7^, VAS score, and Harris score of hip function and postoperative complications at 1, 3, 6, and 12 months were performed between the two groups. In this study, all images used for imaging assessment were repeatedly evaluated and measured by two senior orthopedic surgeons, and the final average value was used for data analysis.

### Statistical methods

The data were processed with IBM SPSS Statistics version 24.0 (https://www.ibm.com/products/spss-statistics), and the measurement data conforming to a normal distribution are expressed as the means ± standard deviations ($\overline{x} \pm s$). Independent samples t tests were used. When the data were nonnormally distributed, the rank-sum test was used. The count data were tested with the *χ*^*2*^ test, and the difference was considered statistically significant at *P* < 0.05.

## Results

All 109 patients were followed up without significant loss to follow-up. The follow-up data, including clinical and imaging assessment results, were complete and reliable and can be used for analysis, and none of them experienced serious complications such as infection, incision nonhealing, deep vein thrombosis of the lower extremity, fracture or dislocation around the joint prosthesis, etc. The complete compliance rate of acetabular cup prostheses in the AI group was 90.9% (50/55), and that of femoral stem prostheses was 87.3% (48/55); in the 2D group, 72.2% (39/54) of acetabular cup prostheses were fully compliant, and the rate of femoral stem prostheses was 66.7% (36/54); the difference was statistically significant (36/54). In the 2D group, the complete compliance rate of the acetabular cup prosthesis was 72.2% (39/54), and the complete compliance rate of the femoral stem prosthesis was 66.7% (36/54); the complete compliance rate of the acetabular cup prosthesis and femoral stem prosthesis in the AI group was significantly greater than that in the 2D group, and the difference was statistically significant (*P* < 0.05). See Table 1. The operation time, degree of intraoperative bleeding, hemoglobin difference, and length of hospitalization in the AI group were significantly shorter than those in the 2D group (*P* < 0.05). See Table 2. The difference in limb length after surgery in the AI group was significantly lower than that in the 2D group (*P* < 0.05), whereas the differences in the anterior tilt angle, abduction angle, and bilateral femoral eccentricity distance between the two groups were not statistically significant (*P* > 0.05). See Table 3. There was no statistically significant difference in the postoperative VAS scores between the two groups (*P* > 0.05). The Harris scores of hip function in the AI group were significantly greater than those in the 2D group at 1, 3, and 6 months postoperatively (*P* < 0.05), and the Harris scores of the two groups were roughly similar at 12 months postoperatively. See Table 4.

**Table 1:** Comparison of the actual prosthetic model and preoperative planning model between the two groups.

	n	Acetabular cup prosthesis*					Femoral stem prosthesis*				
		− 2	− 1	0	+ 1	+ 2	− 2	− 1	0	+ 1	+ 2
AI group	55	0	3	50	2	0	0	2	48	5	0
2D group	54	3	4	39	4	4	4	4	36	7	3
*χ*^*2*^		6.351	6.546								
*P* value		0.012	0.011								

*Prosthesis model compliance is divided into five levels: “− 2, − 1, 0, + 1, + 2”, where “0” represents full compliance. “ + ” means that the actual intraoperative prosthesis model is larger than the preoperative planned model, and “−” means that the actual intraoperative prosthesis model is smaller than the preoperative planned model.

**Table 2:** Perioperative observation index ($\overline{x} \pm s$).

	n	Surgical time (min)	Intraoperative bleeding (mL)	Hemoglobin difference (g/L)	Length of hospitalization (d)
AI group	55	114.24 ± 21.73	172.15 ± 38.89	25.73 ± 8.70	7.36 ± 1.63
2D group	54	145.72 ± 32.42	211.30 ± 49.08	34.43 ± 12.00	9.42 ± 2.25
*t*		− 5.966	− 4.621	− 4.341	− 5.486
*P* value		0.000	0.000	0.000	0.000

**Table 3:** Postoperative imaging index ($\overline{x} \pm s$).

		Forward tilt angle (°)	Abduction angle (°)	Bilateral femoral eccentricity difference (mm)	LLD (mm)
AI group	55	13.63 ± 3.01	42.95 ± 5.70	5.09 ± 3.32	4.05 ± 1.67
2D group	54	12.60 ± 2.48	41.28 ± 6.85	6.27 ± 4.26	5.94 ± 1.65
*t*	1.943	1.388	− 1.621	− 5.935	
*P* value		0.055	0.168	0.108	0.000

**Table 4:** Comparison of postoperative hip function (Harris score) ($\overline{x} \pm s$).

	n	PO 1 month	PO 3 months	PO 6 months	PO 12 months
AI group	55	67.51 ± 5.31	77.60 ± 4.43	89.07 ± 4.78	94.98 ± 2.31
2D group	54	63.76 ± 4.73	73.26 ± 5.69	84.69 ± 3.72	94.07 ± 2.54
*t*		3.891	4.452	5.341	1.952
*P* value		0.000	0.000	0.000	0.054

## Discussion

THA has greatly improved the quality of life of patients with end-stage hip diseases. With the development of science and technology and improvements in living standards, the public’s expectations and requirements for functional rehabilitation after THA are increasing, which makes operators increasingly strict in the selection of prostheses and the accuracy of placement^8,9^. The choice of prosthesis and the precision of placement are becoming increasingly stringent. Accurate preoperative planning helps to obtain information about the patient’s reconstructive outcome, improves the quality of surgery, and reduces the occurrence of postoperative complications. At present, the most widely used preoperative planning method is the 2D X-ray film template measurement method; because the 2D template lacks three-dimensional visual stereo analysis during measurement, it is easy to ignore the anatomical details, and it is subject to a large influence of the subjective factors of the measurer, so it is not possible to accurately judge the angle and depth of the joint prosthesis, which ultimately leads to a high incidence of intraoperative and postoperative complications^10^. This ultimately leads to a high incidence of intraoperative and postoperative complications. Although medical robotic-assisted or intraoperative navigation systems can provide relatively accurate preoperative planning, they are time-consuming, costly, involve a long learning curve, and are not yet an ideal technology for routine application^11,12^. With the advancement and development of science and technology, preoperative planning for THA has gradually shifted from the traditional 2D template method to three-dimensional template planning based on CT scans for more accurate placement of hip components^13^. The following is a summary of the results of this study.

AI-HIP is a 3D program for image processing based on CT data, which has the unique advantage of accurately identifying and reconstructing a patient’s individualized skeletal model for accurate preoperative planning by combining AI deep learning technology and G-NET neural network technology with medical big data^14^. Studies have shown that the use of computerized navigation systems is able to obtain a more satisfactory position of the acetabular cup and femoral stem as well as a range of limb length discrepancies^15^. The use of a computerized navigation system has been shown to result in satisfactory acetabular cup and femoral stem positions and a range of limb length discrepancies. One study reported that CT-based computer-assisted navigation resulted in an average limb length discrepancy of 3.0 mm in patients after surgery^16^. In this study, the AI-HIP had a complete accuracy of 90.9% for the acetabular cup and 87.3% for the femoral stem, which was significantly better than that of the 2D template group; moreover, the postoperative LLD in the AI group was 4.05 ± 1.67 mm, which was no lower than the results of previous studies using instruments or navigation. With the 3D reconstruction technique, the AI-HIP software was able to accurately calculate the transverse diameter of the acetabulum, an ability that is critical for intraoperative sizing of the acetabular component. In addition to the high degree of consistency in acetabular prosthesis planning, the AI-HIP also demonstrated many advantages in performing femoral prosthesis planning, which may be attributed to the fact that when conventional 2D templates are performed at the 2D level on radiographs, rotation of the lower extremity is examined at the 2D level on radiographs, the rotation of the lower extremity greatly affects the accuracy of preoperative planning, and the differences in the anterior inclination of the femoral neck in different populations make accurate calculations with the traditional 2D template method difficult. In this study, the perioperative-related indices were also compared and analyzed, and the results revealed that the duration of surgery and perioperative blood loss in the AI group were significantly lower than those in the 2D group, which can be attributed to accurate preoperative planning, which guided the number of intraoperative osteotomies as well as the depth of expansion of the socket and medulla oblongata and reduced the number of attempts of the prosthetic components in the operation, thus accelerating the surgical process.

Compared with previous 2D and 3D studies, AI-HIP is based on deep learning of the database, which has innovations and advantages such as simple operation, strong visualization and intelligent planning. With the continuous development of this technology, more diverse prostheses will be available for selection in the future, which will meet standardization requirements while achieving personalization. There are also limitations in this study: (1) This study is limited to the DePuy Synthes prosthesis, which is a small range of choices; (2) the sample size is small, and the disease influencing factors are only analyzed for ischemic necrosis of the femoral head, without further subdividing the difference in the accuracy of the software in different diseases; (3) there are a small number of imaging parameters to be evaluated, and only the angle of anterior inclination, abduction angle, bilateral femoral eccentricity and LLD are evaluated, and there is no further evaluation of the acetabular center of rotation. A systematic review further identifies critical limitations in current AI applications for joint replacement research: (1) Methodological deficiencies, evidenced by the fact that only 3 of 76% original studies conducted external validation, with most relying solely on internal validation while conflating “AI” and “machine learning” concepts; (2) Data quality limitations, where incomplete or biased datasets restrict model accuracy, compounded by insufficient code transparency; and (3) Application focus imbalance, with disproportionate attention to clinical decision support over more feasible administrative applications^7^. As echoed in the work of Jaret et al., these findings underscore the imperative for rigorous external validation and clinically viable implementation. Future research should focus on expanding implant databases, integrating multimodal data streams, and addressing unresolved challenges such as implant size prediction to advance standardized AI adoption in orthopedics. In the future, this study will continue to expand the number of cases and conduct clinical RCTs to further explore the accuracy of AI technology in different disease applications and to assess its reproducibility in biomechanical planning and its impact on postoperative clinical outcomes^17^.

In conclusion, the use of AI three-dimensional reconstruction technology for the preoperative planning of patients undergoing THA can assist clinicians in completing surgery more quickly and accurately, effectively reduce intraoperative bleeding, lower postoperative LLD, and, to a certain extent, improve the stability of the postoperative hip joint, accelerate patients’ postoperative functional rehabilitation, and improve their degree of satisfaction.

## Data Availability

The datasets generated during and/or analysed during the current study are not publicly available due to the protection of hospital and patient data but are available from the corresponding author on reasonable request.
